# Neural Mechanisms of Selective Attention in Children with Amblyopia

**DOI:** 10.1371/journal.pone.0125370

**Published:** 2015-06-11

**Authors:** Aibao Zhou, Yanfei Jiang, Jianming Chen, Jianlan Wei, Baobao Dang, Shifeng Li, Qiongying Xu

**Affiliations:** 1 School of Psychology, Northwest Normal University, Lanzhou, Gansu, China; 2 Rehabilitation Hospital Center of Gansu, Lanzhou, Gansu, China; 3 State Key Laboratory of Cognitive Neuroscience and Learning, Beijing Normal University, Beijing, China; University of Montreal, CANADA

## Abstract

Previous studies have indicated that amblyopia might affect children's attention. We recruited amblyopic children and normal children aged 9–11 years as study subjects and compared selective attention between the two groups of children. Chinese characters denoting colors were used in the Stroop task, and the event-related potential (ERP) was analyzed. The results show that the accuracy of both groups in the congruent condition was higher than the incongruent condition, and the reaction time (RT) of amblyopic children was longer. The latency of the occipital P1 in the incongruent condition was shorter than the neutral condition for both groups; the peak of the occipital P1 elicited by the incongruent stimuli in amblyopic children was higher. In both groups, the N1 peak was higher in the occipital region than frontal and central regions. The N1 latency of normal children was shorter in the congruent and neutral conditions and longer in the incongruent condition; the N1 peak of normal children was higher. The N270 latencies of normal children in the congruent and neutral conditions were shorter; the N270 peak was higher in parietal and occipital regions than frontal and central regions for both groups. The N450 latency of normal children was shorter; in both groups, the N450 average amplitude was significantly higher in the parietal region than central and frontal regions. The accuracy was the same for both groups, but the response of amblyopic children was significantly slower. The two groups showed differences in both stages of the Stroop task. Normal children showed advantages in processing speed on both stimulus and response conflict stages.Brain regions activated during the Stroop task were consistent between groups, in line with their age characteristics.

## Introduction

Amblyopia is a common visual impairment in children. The prevalence of amblyopia in China is [1]. In 2013, a local (Lanzhou) vision screening was completed with 12,361 primary and secondary students and there were 445 amblyopic students with an incidence of 3.6% [2]. This disease is congenital or results from interference by certain factors that cause insufficient light stimulus to enter the eyes at a critical period in visual development. As a result, the lack of effective stimulation of visual cells deprives the macula lutea of the opportunity for forming clear images; that is, so-called visual deprivation. In addition, an imbalance between visual inputs from the two eyes leads to competition between clear images and fuzzy objects (binocular competitive inhibition). One of the above two factors or both together can result in deterioration of monocular or binocular vision [3], and the best-corrected visual acuity is lower than that of the normal population at the same age [4]. Amblyopia is mainly a functional deficit of the visual cortex [5–8]. Amblyopia can lead to poor vision, as well as multiple types of functional visual deficits, such as impairments in contrast sensitivity, vernier acuity, contour integration, and binocular vision [9]. Amblyopia not only seriously damages children's visual function, but also affects perception, attention, and other individual psychological components, often leading to a range of psychological and behavioral problems such as inattention, and decreased ability at learning and social adaptation[10]. Currently, the study of amblyopic children is mainly focused on perception disorders and rehabilitation; little research is conducted on relatively higher-level cognitive functions. This study is a comparative study of selective attention between amblyopic children and normal children using event-related potentials (ERP) and a Stroop task. Chinese color words served as experimental material.

The Stroop effect refers to the phenomenon of interference between the color and the meaning of the same stimulus. Early theorists hypothesized that interferences were caused by response competition in the late processing period[11]. The relative speed of processing theory suggested that people have different processing speeds on the two stimuli dimensions (color and word). Reading a word is always faster than naming a color. If the word is consistent with the color, then it will promote the naming of the color; if not, it will interfere with the naming of the color. In consideration of the function of attention during processing, automaticity theory distinguished the concepts of automatic and controlled processing. During the task, reading a word is automatic processing and does not require attention while naming a color is controlled processing and requires attention. The theory of perceptual coding emphasized that interference happens at the coding phase in the early processing period, during which the perceptual coding of the color is impeded by the mismatching information of the color word [11]. Logan’s parallel processing model regards the Stroop effect as the process of collecting evidence for decision-making. The processing speed of a stimulus in each dimension is determined by its weight, which affects the contribution of each dimension to decision-making [12]. The theory of parallel distributed processing (PDP) is a further refinement of the above theories. The PDP system includes many interconnected modules and each module has many simple and interconnected processing units. Each unit is in charge of receiving input from other units and providing output to the other units. The color and word information proceeds in two access pathways with the same response mechanisms. During the processing, there will be information interaction (interference and promotion) [13, 14]. The activation level of each unit is affected by the total input weight while attention can improve the sensitivity of access pathways to inputs. The system can also convey learning and training to produce a certain response to a certain situation. Since PDP theory still did not comprehensively interpret the Stroop effect, in 2003, Robert et al. proposed tectonic theory. This theory proposed four different types of contexts influencing Stroop effects: the background of presented stimulus, the number and magnitude of the stimulus, the congruity effect, and the task effect. The effort that the participants made on the information selection may be neutralized by new information [15]. Current studies suggest that the generation of the Stroop interference effect is the result of the combined effects of stimulus and response conflicts [16]. Stimulus conflict is an interference between the task-related dimension (color) caused by target stimulation and the non-task-related dimension (meaning) caused by distraction stimulation. The similarity of color and meaning determines the degree of the interference effect. The more similarities they have, the less the interference effect will be [17, 18]. Response conflict is the competition between two incompatible response trends before the response which further improves the interference effect [19]. It should also be made clear early in the paper what brain mechanisms are usually studied using the Stroop task, such as executive functions[16, 20], cognitive flexibility[21, 22], processing speed[23], conflict management[24–26], attention etc.

The Stroop test is one of the most commonly used tasks in the study of visual selective attention. The Stroop effect first appears in an individual during the lower grades in elementary school[11]. Its interference declines with the development of reading ability over the years [27], and increases again by the time the individual approaches the age of 60 years. Development of the Stroop effect in children involves a complex process. The interference effect results from the interaction of color recognition of stimuli in the early phase with response selection in the later phase, while a promoting effect occurs in the response selection phase [28]. Studies have found that in the Stroop task, the amount of interference of Chinese characters, Pinyin, and English words decreases successively and the PDP model provides a good explanation of the experimental results [29]. The naming response to color information of Chinese characters whose semantic properties contain color meaning is much faster (color patches, color words) than to those whose semantic properties do not contain color meaning [30]. The Stroop effect is the results of the combined effect of stimulus and response conflicts and cannot be studied further by behavioral experiments. Event-related potential (ERP), which uses the millisecond as its timing unit, has a high degree of time accuracy. Its combination with the Stroop paradigm can accurately investigate the variation between these two conflicts. This combination enables this study to further examine the different expressions of the Stroop effect on amblyopic children and normal children. In other words, it helps further illustrate whether there is any difference in the stages of stimulus and response conflicts between amblyopic children and normal children.

ERP is an imaging technique that will not damage brain cognitive nerves. Its potential variation is a time-related brain electrical activity of human beings’ physical or mental activities. Such variation is recorded at the scalp and separated from EEG by signal filtration and superposition. The early components of ERP usually refer to the potential variation within 200 ms after stimulation and are mainly associated with the physical properties (intensity, type, frequency, etc.) such as hearing P50 and vision P1. The late components of ERP are mainly related to the processing of human beings’ consciousness or cognitive psychological functions (attention, memory, etc.) such as P300 and N400. Early visual components of ERP typically include C1, and visual P1 and N1, which mainly reflect an individual’s visual perceptual processing of stimuli [31–35]. C1 is the first apparent ERP component. It is very sensitive to stimuli physical properties (contrast, brightness) and is not related to attention [32]. P1 appears after C1, mainly at the occipital regions on both sides, and is associated with attention. When the supraliminal visual stimulus emerges, the stimulus that attracts the participants’ attention will induce a bigger P1 wave in the cerebral cortex [31]. The visual N1 appears after P1 and includes several sub-components. The earliest sub-component is the wave in the frontal lobe 100–150 ms from stimulus onset. The back of the head has at least two sub-components coming from the parietal-occipital region and the peak appears 150–200 ms from stimulus onset. The sub-components of the occipital region reflect a distinguishing process [31, 34]. The N270 was first found and named by the Chinese scholar Wang Yuping [36]. The N270 is elicited by conflicting stimuli and reflects the cerebral processing of conflict [36, 37]. When the information of the compared parts conflicts, the N270 can be recorded 270 ms after the appearance of the second information. The N450 is the specific brain electrical component of the Stroop task and is mainly located in the medial and anterior of the cephalic region [38, 39]. The N450 is related to a broad form of conflict monitoring by the brain. It is sensitive to both response and non-response conflicts [16, 38].

Selective attention means that an individual chooses to attend to one stimulus from two or more stimuli and neglects other stimuli. Selective attention has great significance for individuals. It allows the individual to make full use of limited mental resources within a short period of time for processing stimuli or events that are crucial to the individual’s survival [40]. The development of selective attention in children shows a constantly developing trend, and is associated with cognitive development [41, 42]. The visual selective attention ability of children at the age of 7–12 years is not maturely developed, and the main underlying mechanism involves top-down regulation [43, 44]. Our study found that there were notable differences between normal children and amblyopic children in terms of attention quality: amblyopic children had the same attention broadness and attention allocation capability, but showed significantly lower attention transfer and stability [45]. No relevant studies focusing on the existence of differences in selective attention between amblyopic children and normal children have been reported. We hypothesized that: (1) the selective attention capability of amblyopic children is lower than that of normal children; and (2) there are differences in the peak amplitude and latency of ERP components between amblyopic children and normal children.

## Methods

### Ethics Statement

The experiment was conducted after obtaining Institutional Review Board approval from the School of Psychology at Northwest Normal University. All the parents of children participants gave informed written consent before testing began.

### Participants

Both the experimental group and the control group included a convenience sample. Amblyopic children, who were receiving outpatient treatment at the Low-vision Rehabilitation Department of the Gansu Provincial Rehabilitation Center Hospital and aged 9–11 years, were included in the experimental group. Normal children aged 9–11 years were selected from an elementary school in Lanzhou for the control group. All participants were right-handed and without other physical or mental illnesses.

This study enrolled 12 amblyopic children, including six males and six females, with a mean age of 10.78 ± 1.15 years. All amblyopic children had mild amblyopia (their binocular corrected visual acuity reached 4.9/0.8 to 4.8/0.6). Eleven normal children with a mean age of 11.47 ± 1.31 years were enrolled in this study, including six males and five females, and all had normal binocular vision.

### Measures

In this experiment, a 2 (participant type: normal and amblyopic children) × 3 (incentive condition: congruent, neutral, and incongruent) mixed design was employed. Participant type was a between-subject variable and incentive condition was a within-subject variable. Reaction time, accuracy, peak value of ERP waveform (the average amplitude), and latency were dependent variables.

In accordance with the experimental materials proposed by Danling Peng *et al*[28], two Chinese color characters, ‘红’ (meaning red in English) and ‘绿’ (meaning green in English) were used as experimental material, and two unrelated Chinese characters (‘涂’ and ‘华’) were used as control material. All characters were written in two colors, and the average frequency of color characters and unrelated characters was matched. All Chinese characters were presented in ‘Song’ font with a font size of 28, and were displayed at the center of a 17-inch monitor screen.

The tests were performed in an EEG laboratory, and participants responded to the color of the presented Chinese characters by pressing the corresponding keys. E-prime 2.0 was used in the experiment to present and record the data. In the tests, ‘1’ was recorded when the color of a Chinese character was identified correctly and ‘0’ was recorded when a wrong answer was provided. The reaction time from the presence of the Chinese character to the pressing of corresponding key was also recorded. Participants placed the index fingers of each hand on the keys for ‘1’ and ‘4’ on the keyboard, and a colored sticker (corresponding to a color) was pasted on each of these two keys. Participants had a short practice session before the formal test to help them to establish a corresponding relationship between the colors and responses[28].

In the formal tests, the participants sat in a quiet room with soft lighting. The background of the display screen for stimulus presentation was gray, the fixation point was black, and Chinese characters colored either red or green were presented in the center of the screen. Participants sat in a comfortable position at a distance of 85 cm to the screen, and looked for stimuli at the center of the screen with a visual angle of 2° × 2°. Prior to the test, the investigator read the instructions to the participants to make sure they fully understood the experimental requirements. Participants then pressed the space bar to begin the test.

**Fig 1:**
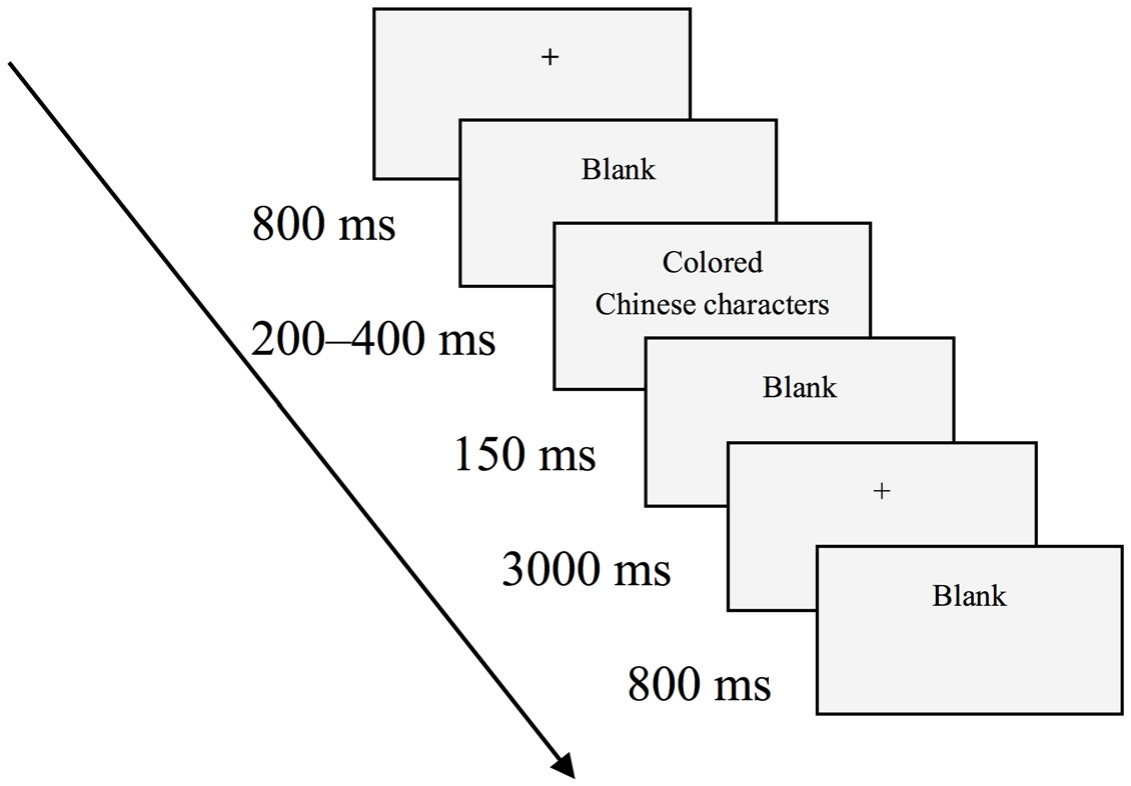
ERP Experimental Flow Chart. The procedure for each block was as follows. Firstly, on a gray screen, a black "+" was presented for 800 ms, followed by a random blank screen for 200–400 ms. Next, a character (in a pseudo-random sequence after matching the characters and colors) was presented for 150 ms and the participant was asked to name the color of the Chinese character by pressing the corresponding key. Subsequently, after stimulus presentation, a blank screen was presented for 3000 ms.

The test consisted of five blocks, and each block included 48 trials (16 each of congruent, incongruent, and neutral stimulus trials). The procedure for each block was as follows. Firstly, on a gray screen, a black "+" was presented for 800 ms, followed by a random blank screen for 200–400 ms. Next, a character (in a pseudo-random sequence after matching the characters and colors) was presented for 150 ms and the participant was asked to name the color of the Chinese character by pressing the corresponding key. Subsequently, after stimulus presentation, a blank screen was presented for 3000 ms (Fig 1). Color stickers on the two keys were evenly distributed; key "1" was labeled red and key "4" labeled green for half of the participants, and key "1" was labeled green and key "4" labeled red for the other half.

### ERP Data Collection and Offline Data Analysis

In this study, EEGs were recorded using a MacPro workstation (EGI, Eugene, OR, US), an Amp300 amplifier, and a 64-channel EEG cap. Electrode positions were assigned using the 10–20 system as a reference and Cz served as a reference electrode for simultaneous recording of horizontal and vertical electrooculography (EOG). All scalp resistance of the electrodes was maintained below 50 KΩ, band pass was 0.1–100 Hz, and the sampling rate was 250 Hz. The recorded EEG data were filtered using 0.1 Hz high-pass and 30 Hz low-pass filters. Continuous EEG and behavioral data were simultaneously recorded. The data in a 200 ms time window prior to stimulus presentation were used as a baseline and the data in an 800 ms time window after stimulus presentation were used for analysis. If the signal change from an electrode exceeded 200μV, then the electrode was marked as false. If more than 10 electrodes in a trial were labeled false, then the trial data were removed from the subsequent analysis. When the horizontal EOG signal changed more by than 140 μν or the vertical EOG signal changed by more than 55 μν, the data for the trial were excluded from further analysis. The data from a false electrode were replaced with the data from nearby electrodes using the interpolation algorithm’ instead.

The waveforms of each group were superimposed to generate three types of ERP waveforms (congruent stimuli, neutral stimuli, and incongruent stimuli). Based on the distribution of ERP topographic maps in previous studies [28, 46, 47] and the present study, three representative electrodes (F3, FZ, F4; C3, CZ, C4; P3, PZ, P4; O1, OZ, O2) were chosen for each of four brain regions (frontal, central, parietal, and occipital regions). The mean peak latency, and average amplitude of ERP waveforms for the different stimulus conditions were subjected to statistical analyses.

## Results

### Behavioral Results

The reaction time (RT) and accuracy of normal children and amblyopic children in the three Stroop task conditions are shown in Table 1 (S1 Data).

**Table 1:** Reaction time (RT, s) and accuracy(ACC,%) of normal and amblyopia children in Stroop task.

	congruent		neutral		Incongruent	
	RT	ACC	RT	ACC	RT	ACC
Normal children (*n* = 11)	0.48±0.14	0.89±0.16	0.48±0.15	0.84±0.17	0.47±0.15	0.79±0.16
amblyopia children (*n* = 16)	0.77±0.45	0.87±0.10	0.77±0.45	0.86±0.08	0.76±0.36	0.85±0.09

A repeated measures analysis of variance (ANOVA) of the 2 (groups: normal group or amblyopia group) × 3 (conditions: congruent, incongruent, or neutral) of the RT and accuracy was conducted for normal children and amblyopic children for the three Stroop task conditions.

The ANOVA of the accuracy revealed a significant primary effect of the stimulus condition (*F* (2,40) = 3.59, *p* <0.05, *η* ^2^ = 0.15), as evidenced by data showing that the accuracy in the congruent condition was significantly higher than the incongruent condition (*p* < 0.05). The differences in the accuracy between the congruent and neutral conditions and between the incongruent and neutral conditions were not statistically significant. The primary effect of group was not significant (*F* (1, 20) = 0.49, *p* >0.05, *η* ^2^ = 0.02) and the interaction between group and condition was not significant (*F*(2,40) = 0.593, *p* >0.05, *η* ^2^ = 0.02). The RT ANOVA indicated a significant primary effect of group (*F*(1, 20) = 1.438, *p* = 0.05, *η* ^2^ = 0.06), as evidenced by data showing that the RT of amblyopic children was significantly longer than that of normal children. The interaction between group and condition was not significant(*F* (2, 40) = 0.96, *p* >0.05, *η* ^2^ = 0.05). (The statistical results of the data is from the S1 Data)

### ERP Analysis

The waveforms of each participant group were superimposed to generate three types of ERP waveforms (congruent stimuli, neutral stimuli, and incongruent stimuli). Based on the average data of ERP from previous studies [28, 46, 47] and the present study, three representative electrodes in each of the four brain regions (frontal, central, parietal, and occipital regions) were selected (F3、FZ、F4、C3、CZ、C4、P3、PZ、P4、O1、OZ、O2). The mean peak latency and average amplitude of ERP waveforms for the different stimulus conditions were subject to a repeated measures ANOVA and the data were corrected using the Greenhouse—Geisser method.

**Fig 2:**
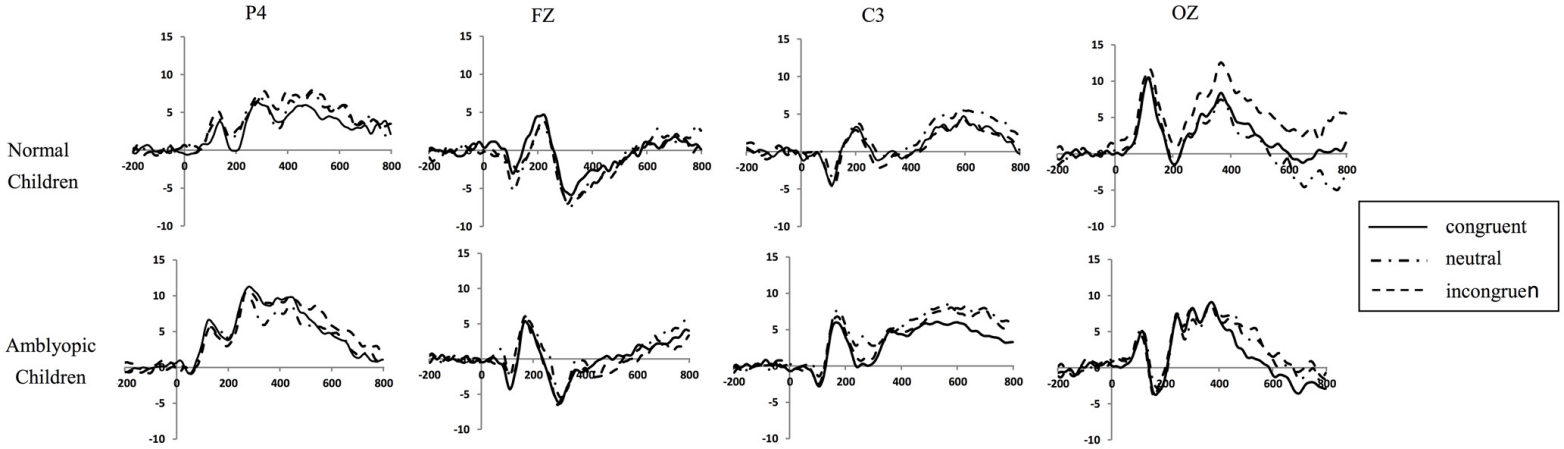
ERP basic waveform of three conditions Stroop. P4, FZ, C3 and OZ change between the normal and amblyopia chilaren in the ERP basic waveform of three conditions Stroop (The statistical results of the data is from the S2–S9 Data).

We chose the peak (average amplitude) and latency of the occipital P1, the frontal, central, and occipital N1, the frontal, central, parietal, and occipital N270, and the central and parietal N450 for further analysis. The time window was 70–180 ms after stimulus presentation for P1 analysis, 70–150 ms after stimulus presentation for the frontal and the central N1, 130–250 ms for the occipital N1, and 200–400 ms for N270. N450 had no obvious peak; therefore, the average amplitude was analyzed within the time window 350–550 ms after the stimulus presentation. The overall average ERP waveforms of both groups for the three conditions of the Stroop effect are shown in Fig 2.

#### Early stages of stimulus conflict

**Fig 3:**
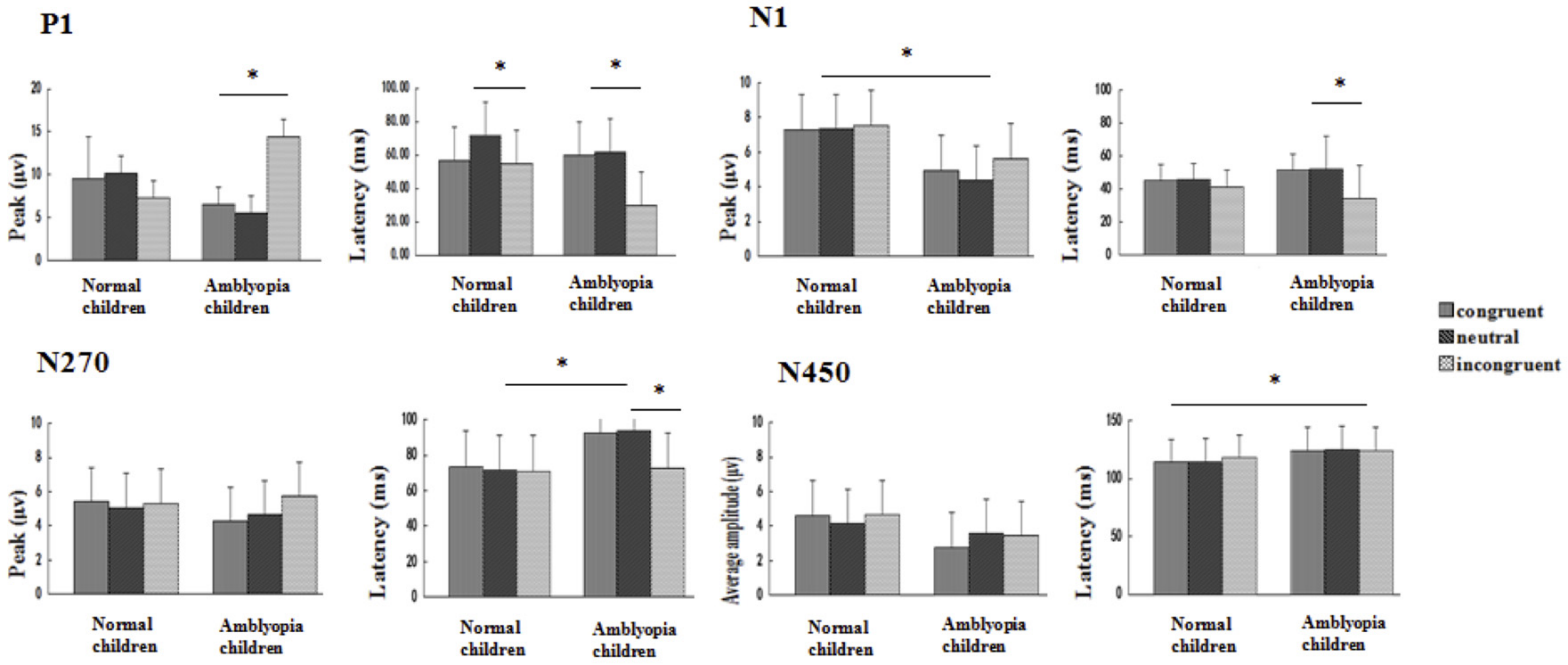
ERP peak (average amplitude) and latency of repeated measures ANOVA. **P1, N1, N270 and N450 change between the normal and amblyopia children in the components of ERP of three conditions Stroop.** Error bars indicate standard errors (The statistical results of the data is from the S2–S9 Data). *p < 0.05.”

We conducted a 2 (groups: normal group or amblyopia group) × 3 (conditions: congruent, incongruent, and neutral) ANOVA of peak and latency of the occipital P1. Peak analysis revealed a significant primary effect of condition (*F*(2, 42) = 5.34, *p* <0.05, *η* ^2^ = 0.20). The Bonferroni method was used for multiple comparisons and revealed that the peak elicited by the incongruent condition was significantly higher than the peak elicited by the congruent and neutral conditions (*p* < 0.05). The difference in the peak value induced by the congruent and neutral conditions was not statistically significant (*p* > 0.05). The primary effect of group was not significant (*F*(1, 21) = 0.02, *p* >0.05, *η* ^2^ = 0.00). The interaction between group and condition was significant (*F*(2, 42) = 11.54, *p* <0.001, *η* ^2^ = 0.35). Simple-effects analysis showed that the peak differences between the three conditions in normal children did not reach statistical significance (*p* > 0.05). In amblyopic children, the peak elicited in the incongruent condition was significantly higher than those elicited in the congruent and neutral conditions (*p* < 0.001); and the peak differences between the congruent and neutral conditions were not statistically significant (*p* > 0.05). In addition, the peak elicited in the incongruent condition in amblyopic children was significantly higher than that of normal children (*p* < 0.05), and the differences in the peaks elicited in the congruent and neutral conditions between amblyopic children and normal children were not statistically significant (*p* > 0.05)(Fig 3)(The statistical results of the data is from the S2 Data).

The analysis of P1 latency indicated a significant primary effect of condition (*F*(2, 42) = 7.28, *p* <0.05, *η* ^2^ = 0.25). Multiple comparison tests using the Bonferroni method showed that the latency in the incongruent condition was significantly shorter than in the neutral condition (*p* < 0.001). In addition, the differences in latency between the neutral and congruent conditions and between the congruent and incongruent conditions were not statistically significant (*p* > 0.05). The primary effect of group was not significant (*F*(2, 42) = 1.83, *p* >0.05, *η* ^2^ = 0.08) and the interaction between condition and group was not significant (*F*(2,42) = 2.28, *p* >0.05, *η* ^2^ = 0.98) (Fig 3)(The statistical results of the data is from the S3 Data).

A repeated measures ANOVA of the N1 peak and latency was conducted using 2 (groups: normal group and amblyopia group) × 3 (conditions: congruent, incongruent, and neutral) × 3 (brain areas: frontal, central area, occipital region). The N1 peak analysis showed a significant primary effect of brain region (*F*(2, 42) = 5.53, *p* <0.05, *η* ^2^ = 0.20). Multiple comparison tests using the Bonferroni method showed that the peak value obtained from the occipital electrodes was significantly higher than the frontal and central regions (*p* < 0.05), and the peak difference between the frontal and central regions was not statistically significant (*p* > 0.05). The primary effect of group was significant (*F*(1, 21) = 6.95, *p* <0.05, *η* ^2^ = 0.24), as evidenced by the fact that the peak of amblyopic children was significantly higher than that of normal children. The primary effect of condition was not significant (*F*(2, 42) = 1.21, *p* >0.05, *η* ^2^ = 0.55). The interactions between condition and group, and between brain region and group were not significant (*F*(2, 42) = 0.56、0.01, *p* >0.05, *η* ^2^ = 0.26、0.00). The interactions between condition and brain region were not significant (*F*(4, 48) = 0.47, *p* >0.05, *η* ^2^ = 0.02). The interactions among condition, brain region, and group were not significant (*F*(4, 84) = 0.32, *p* >0.05, η^2^ = 0.01) (Fig 3)(The statistical results of the data is from the S4 Data).

The analysis of N1 latency showed a significant primary effect of condition (*F*(2, 42) = 30.89, *p* <0.001, *η* ^2^ = 0.59). Multiple comparison tests using the Bonferroni method showed that the N1 latency in the incongruent condition was remarkably shorter than that in the congruent and neutral conditions (*p* < 0.001), and the latency in the congruent condition was remarkably shorter than that in the neutral condition (*p* < 0.05). The primary effect of brain region was significant (*F*(2, 42) = 7.29, *p* <0.001, *η* ^2^ = 0.25). The N1 latency for the occipital electrodes was significantly longer than the frontal and central areas (*p* < 0.05), and the latency for the frontal and central electrodes was not significantly different (*p* > 0.05). The primary effect of group was not significant (*F*(1, 21) = 1.15, *p* >0.05, *η* ^2^ = 0.05). The interaction between condition and group was significant (*F*(2, 42) = 12.38, *p* <0.01, *η* ^2^ = 0.37). Simple-effects analysis showed that the difference in latency between the three conditions in normal children was not statistically significant, while the latency in amblyopic children in the incongruent condition was shorter than in the congruent and neutral conditions (*p* < 0.001), and the latency in the congruent condition was shorter than in the neutral condition (*p* < 0.05). Under the congruent and neutral conditions, the N1 latency in amblyopic children was longer than that of normal children (*p* < 0.01), and the N1 latency in amblyopic children in the incongruent condition was shorter than that of normal children (*p* < 0.05). The interaction between brain region and group was not significant (*F*(2, 42) = 7.29, *p* >0.05, *η* ^2^ = 0.25). The interactions between condition and brain region were not significant (*F*(4, 84) = 0.27, *p* >0.05, *η* ^2^ = 0.01). The interactions among condition, brain region, and group were not significant (*F*(4, 84) = 0.90, *p* >0.05, *η* ^2^ = 0.04) (Fig 3)(The statistical results of the data is from the S5 Data).

The N270 peak and latency were subjected to a 2 (groups: normal group or amblyopia group) × 3 (conditions: congruent, incongruent, or neutral) × 4 (brain regions: frontal, central area, parietal, or occipital region) ANOVA. The analysis of the N270 peak showed a significant primary effect of brain region (*F*(3, 63) = 34.26, *p* <0.001, *η* ^2^ = 0.62). The multiple comparison tests using the Bonferroni method showed that the peaks from parietal and occipital electrodes were significantly higher than those in the frontal and central regions (*p* < 0.001). The peak differences between the parietal and occipital electrodes and between the frontal region and central electrodes were not statistically significant (both *p* > 0.05). The primary effect of condition was not significant (*F*(2, 42) = 1.88, *p* >0.05, *η* ^2^ = 0.08) and the primary effect of group was also not significant (*F*(1, 21) = 1.18, *p* >0.05, *η* ^2^ = 0.01). The interaction between condition and group was not significant (*F*(2, 42) = 1.86, *p* >0.05, *η* ^2^ = 0.08), the interaction between brain region and group was not significant (*F*(3, 63) = 0.23, *p* >0.05, *η* ^2^ = 0.01), and the interactions between condition and brain region were not significant (*F*(6, 126) = 0.61, *p* >0.05, η^2^ = 0.02), and among condition, brain region, and group were not significant (*F*(6, 126) = 1.64, *p* >0.05, *η* ^2^ = 0.07) (Fig 3)(The statistical results of the data is from the S6 Data).

The analysis of N270 latency showed a significant primary effect of condition (*F*(2, 42) = 30.51, *p* <0.001, *η* ^2^ = 0.59). Multiple comparison tests using the Bonferroni method showed that the latency in the incongruent condition was shorter than in the congruent and neutral conditions (*p* < 0.001), and the difference in latency between the congruent and neutral conditions was not statistically significant (*p* > 0.05). The primary effect of group was significant (*F*(1, 21) = 10.39, *p* <0.01, *η* ^2^ = 0.33), and the N270 latency in normal children was shorter than that of amblyopic children (*p* < 0.01). The primary effect of brain region was not significant (*F*(11, 231) = 0.74, *p* >0.05, *η* ^2^ = 0.03). The interaction between condition and group was significant (*F*(2, 42) = 23.37, *p* <0.001, *η* ^2^ = 0.52). Simple-effects analysis revealed that the differences in latency in normal children for the three conditions were not statistically significant. For amblyopic children, the latency in the incongruent condition was shorter than in the congruent and neutral conditions (*p* < 0.001), and the difference in latency between the congruent and neutral conditions was not statistically significant (*p* > 0.05). The N270 latency of amblyopic children was longer than that of normal children under the congruent and neutral conditions (*p* <0.01), and the difference in latency between amblyopic children and normal children in the incongruent condition was not statistically significant (*p* > 0.05). The interaction between brain region and group was not significant (*F*(3, 63) = 0.57, *p* >0.05, *η* ^2^ = 0.02). The interactions between condition and brain region were not significant (*F*(6, 126) = 1.47, *p* >0.05, *η* ^2^ = 0.06). The interactions among condition, brain region, and group were not significant (*F*(6, 126) = 1.88, *p* >0.05, *η* ^2^ = 0.08) (Fig 3)(The statistical results of the data is from the S7 Data).

#### Response-conflict stage

The N450 average amplitude and latency were subjected to a 2 (groups: normal group or amblyopia group) × 3 (conditions: congruent, incongruent, or neutral) × 3 (brain regions: frontal, central, or parietal regions) ANOVA. The analysis of the N450 average amplitude showed that the primary effect of brain region was significant (*F*(2, 42) = 20.03, *p* <0.001, *η* ^2^ = 0.48). Multiple comparison tests using the Bonferroni method showed that the average amplitude for the parietal electrodes was significantly higher than the central and frontal regions (*p* < 0.05), and the average amplitude for the central electrodes was significantly higher than the frontal area (*p* < 0.05). The primary effect of condition was not significant (*F*(2, 42) = 0.18, *p* >0.05, *η* ^2^ = 0.01). The primary effect of group was not significant (*F*(1, 21) = 0.89, *p* >0.05, *η* ^2^ = 0.04). The interactions between condition and group, and brain region and group were not significant (*F*(2, 42) = 0.63、0.10, *p* >0.05, *η* ^2^ = 0.02、0.01). The interactions between condition and brain region, and between condition, brain region, and group were not significant (*F*(4, 84) = 0.10、0.34, *p* >0.05, *η* ^2^ = 0.01、0.01) (Fig 3)(The statistical results of the data is from the S9 Data).

The analysis of N450 latency showed that the primary effect of brain region was significant (*F*(2, 42) = 14.48, *p* <0.001, *η* ^2^ = 0.40). Multiple comparison tests using the Bonferroni method showed that the latency for the parietal electrodes was significantly shorter than the central and frontal regions (*p* < 0.05), and the difference in latency between the central and frontal regions was not statistically significant. The primary effect of group was significant (*F*(1, 21) = 6.29, *p* <0.05, *η* ^2^ = 0.23), and the N450 latency of normal children was shorter than that of amblyopic children. The primary effect of stimulation condition was not significant (*F*(2, 42) = 0.26, *p* >0.05, *η* ^2^ = 0.01). The interactions between condition and group and between brain region and group were not significant (*F*(2, 42) = 0.45、1.08, *p* >0.05, *η* ^2^ = 0.02、0.04). The interactions between condition and brain region, and between condition, brain region, and group were not significant (*F*(4, 84) = 0.56、1.13, *p* >0.05, *η* ^2^ = 0.02、0.05) (Fig 3)(The statistical results of the data is from the S8 Data).

## Discussion

The behavioral experiments showed that children with amblyopia had a slower selective attention response than normal children, but there was no difference in the accuracy between amblyopic children and normal children. Inadequate light stimulation of the eye and (or) an imbalance in binocular visual input affects the conduction of visual information and ultimately the formation of advanced visual function in amblyopic children [48]. Not only is the best-corrected visual acuity of amblyopic children lower than that of normal children at same age, but they also have less visual perception experience than normal children. Therefore, compared with normal children, although amblyopic children had the same accuracy in the recognition test of Chinese characters and colors, it takes amblyopic children a longer time to identify the stimulus.Amblyopic and normal children did not significantly differ in their responses to the three conditions (congruent, incongruent, and neutral ones), and no typical Stroop effect occurred. The interference of the Stroop effect weakens with increasing age and reading comprehension. The two groups of children were all primary students of grade 4 or 5 with good reading ability and they were quite familiar with the Chinese characters used in the experiment. Thus, the interference of Stroop effect should be weakened. Attention is another factor that affects the Stroop effect. Under the condition of focused attention, the Stroop effect will be reversed (i.e., the reaction time under the incongruent condition is shorter than that under the congruent one) [49, 50]. Perhaps children paid more attention to the more difficult tasks (incongruent condition) and thus the experimental result was affected. Practice can increase the familiarity of materials and tasks and strengthening color-naming pathways and/or restraining the character reading pathway can weaken the Stroop effect. Compared to response conflict, stimulus conflict is prone to practice effects[51]. Before the test, children require practice to build the mapping relationship between the color and the response, which may affect the results of the behavioral experiment. A Stroop task contains stimulus conflict and response conflict[18, 52]; whether the RT difference between the two groups of children reflects one or both types of conflict needed to be investigated further using the ERP data.

The P1 is related to attention [31, 33, 53], and a suprathreshold visual stimulus that captures the participant’s attention can induce a greater P1 wave in the cerebral cortex [31, 33]. This study found that the P1 latency in the incongruent condition was significantly shorter than that in the neutral condition for both groups of participants, and the latency difference between the two groups was not statistically significant. Vision is an active process that is affected by top-down processing (e.g., attention, anticipation, and task detection) [54]. The attention of an individual can be selectively concentrated on the relevant stimuli and thus irrelevant stimuli can be ignored [43, 44, 54]. The integrated schema theory of Neisser proposed that attention is jointly regulated by pre-attention processing and attention processing [55]. The attention and control processing occur in sequence later, and require a certain amount of time and attentional resources. In an incongruent condition, the interference between the meaning and color of the word leads to an increase in the consumption of attentional resources. The participant selectively concentrates a greater amount of attention on the incongruent stimulus. This top-down regulation increases the sensitivity of children to incongruent stimuli and leads to an earlier processing of the stimulation. Studies on the pattern visual evoked potential (P-VEP) [56, 57] have showed that amblyopic children have a delayed P100 with a longer latency and a decreased P100 peak value for the amblyopic eye. This study found that the P1 peak elicited in the incongruent condition in amblyopic children was higher than that of normal children, and also higher than that elicited in the congruent and neutral conditions in amblyopic children. The results of this study differ from previous studies, which may be related to multiple factors such as the stimulation material and comprehensive rehabilitation treatments. P-VEP tests are usually conducted using an alternating checkerboard, and thus the stimulation is congruent. However, in this study, Chinese characters in different colors were used as stimulus material; therefore, there are some differences in the stimulus materials between the three conditions. In addition, because the amblyopic children participating in this study were receiving comprehensive rehabilitation treatment, the difference may be caused by visual function training[58, 59].

The N1 appears after the P1. The N1 components from both sides of the occipital cortex reflect certain detection procedures, reaching a higher peak when the individual is performing a detection task [31, 34]. Among several N1 subcomponents, the peak from the electrodes on the front of the head is activated first, and the peak of the N1 sub-component at the back of head usually appears 150–200 ms after the stimulus presentation. In this study, the time window for the occipital N1 was 130–250 ms after the stimulus presentation. For both groups, the peaks from the occipital electrodes were significantly higher than those in the frontal and central areas, and the latency for the occipital electrodes was significantly longer than frontal and central areas, which was consistent with previous findings. Under all three conditions, the N1 peak of normal children was significantly higher than that of amblyopic children. The N1 latency of normal children was shorter than that of amblyopic children in the congruent and neutral conditions, and was longer than that of amblyopic children in the incongruent condition. In amblyopic children, the precise processing of visual information is affected by X-cell dysfunction in the visual pathway, which may be accompanied by a variety of functional visual impairments [9]. Therefore, the processing depth and the ability of amblyopic children to identify stimuli are not comparable with normal children. Attention is an important factor that is related to the N1 latency [33, 53]. Both the attention filter theory and the attentional resources theory of selective attention assert that the difficulty of a task will have an impact on attention; in particular, perception is more easily influenced by a difficult task at the early stage [60]. Because amblyopic children focus more resources on incongruent stimuli, their N1 latency in an incongruent condition is shorter than that of normal children.

The N270 is a negative component evoked during matching tasks [36]. N270 is evoked by conflicting stimuli and reflects the cerebral processing of conflicts [37]. This study found that the peaks for the parietal and occipital electrodes were significantly higher than the frontal and central regions, consistent with previous studies. The development of functions related to the Stroop task in children mainly occurs in the parietal lobe[46, 61]. Previous studies have observed the conflict event-related potential N270 in school-age children [62]. Its occurrence is controlled by attention and its scalp potential distribution is impacted by the characteristics of attentional stimuli. In addition, the top-down inhibitory regulation of the conflict management system will affect the N270 peak [63, 64]. In this study, the N270 was triggered in both amblyopic children and normal children by their selective attention to the conflict between the meaning and color of the character, and the peak difference between the two subject groups in the three conditions was not statistically significant. This study also found that the N270 latencies in normal children for the congruent and neutral conditions were shorter compared with amblyopic children, whereas the difference was not statistically significant for the incongruent condition. Processing speed can be used to reflect the operating speeds for different cognitive operations. Compared with normal children, amblyopic children were slower at identifying the conflict between the meaning and color of Chinese characters. This result indicates that amblyopic children possess a certain ability to exert inhibitory control on the meaning of a character involved in a conflict, but their ability to inhibit the conflict is inferior to that of normal children.

The STROOP task can trigger a N450 component[38], which reflects monitoring of response conflict and non-response conflict [65]. In a ‘manual’ Stroop task in which participants press keys in response to stimuli, the N450 is evoked mainly in the central-parietal or parietal regions[66]. The N450 activation in the central-frontal part of the scalp may reflect response-conflict monitoring [52]. This study found that the N450 average amplitude was significantly higher in the parietal and central regions than in the frontal region, and the latency for the parietal electrodes was significantly shorter than the central and the frontal regions. Using a method in which participants manually respond by pressing a key, this study has yielded conclusions consistent with previous N450-related studies[38, 52, 66–68]. The development of functions related to the Stroop task occurs mainly in the parietal lobe in childhood, and occurs in the prefrontal cortex only when the individual reaches the age of 18–22 years[46]. The differences in the N450 average amplitude were not statistically significant between normal children and amblyopic children for the three conditions. The N450 latency in normal children was significantly shorter than that of amblyopic children. In this study, participants were children aged 10–11 years, and amblyopic children were able to identify conflicting information, but at a slower rate than normal children.

The behavioral experiment did not have the typical Stroop effect, which might be related to the age, attention, and practice effects in the two groups of children. The early visual components of ERP mainly reflect individuals’ visual perceptions of stimuli. The two groups of children’s P1 and N1 component latencies under the incongruent condition were obviously shorter than those under the congruent or neutral conditions without the typical Stroop effect. All of the children chose to selectively pay more attention to the incongruent stimulus and this top-down adjustment weakened the interference of the Stroop effect. Meanwhile, the influence of improved reading ability and practice also weakened the Stroop effect interference at the stimulus discrimination stage. No typical Stroop effect occurred at the stimulus conflict stage either. The latency of the N270 component under the incongruent condition was shorter than those under the congruent and neutral conditions. N270 generation was controlled by attention. Practice also had impacted the stimulus conflict stage. Influenced by the above reasons, the typical Stroop effect did not occur in the behavioral experiment.

The conflicts in the Stroop task occur at the late stimulus processing stage [69]. The early ERP components (such as N1 and P1) display no notable differences under the incongruent and congruent conditions [66, 70]. The late brain electrical components have been studied in most research. Considering the amblyopic children specifically, this study conducted a complete comparison and analysis of the ERP components of two groups of children from the early stage. At the stage when attention began, amblyopic and normal children showed no significant differences in processing speed or depth. At the stimulus discrimination stage, normal children had the advantage in processing depth, but both groups had advantages and disadvantages in processing speed; the processing speed of amblyopic children under the incongruent condition was faster, while the processing speed of normal children was faster under the congruent and neutral conditions. We found differences between amblyopic children and normal children in both stages. At the stimulus conflict stage, the difference in the two groups of children was reflected in processing speed. Normal children’s processing speed was faster under the congruent and neutral conditions. At the response conflict stage, their difference was still reflected in processing speed. The processing speeds of normal children under these three conditions were all faster than those of the amblyopic children. The brain regions activated during the Stroop task were consistent in amblyopic children and normal children, in line with their age characteristics.

## Conclusion

The accuracy of amblyopic children’s selective attention was the same as that of normal children but their reaction speed was obviously slower than that of normal children.

At the stimulus and response conflict stages, normal children only had a processing speed advantage.

## Supporting Information

S1 Data Behavioral Data (The reaction time and accuracy of normal children and amblyopic children in the three Stroop task conditions.) (SAV)

S2 Data P1 peak (P1 composition peak of ERP data of normal children and amblyopic children in the three Stroop task conditions.) (SAV)

S3 Data P1 latency (P1 composition latency of ERP data of normal children and amblyopic children in the three Stroop task conditions.) (SAV)

S4 Data N1 peak (N1 composition peak of ERP data of normal children and amblyopic children in the three Stroop task conditions.) (SAV)

S5 Data N1 latency (N1 composition latency of ERP data of normal children and amblyopic children in the three Stroop task conditions.) (SAV)

S6 Data N270 peak (N270 composition peak of ERP data of normal children and amblyopic children in the three Stroop task conditions.) (SAV)

S7 Data N270 latency (N270 composition latency of ERP data of normal children and amblyopic children in the three Stroop task conditions.) (SAV)

S8 Data N450 latency (N450 composition latency of ERP data of normal children and amblyopic children in the three Stroop task conditions.) (SAV)

S9 Data N450 average amplitude (N450 composition average amplitude of ERP data of normal children and amblyopic children in the three Stroop task conditions.) (SAV)
